# Being Present in the Silence

**DOI:** 10.4269/ajtmh.21-0805

**Published:** 2021-11-01

**Authors:** Abigail Patterson, Eshana Shah

**Affiliations:** ^1^ Department of Pediatrics, Division of Neonatal-Perinatal Medicine, University of Texas Southwestern Medical Center, Dallas, Texas;; ^2^ Department of Medicine, Division of Hematology and Oncology, University of Illinois at Chicago, Chicago, Illinois

It was 5:30 pm on a Friday on our last clinic day.

We were fourth year medical students, a few weeks shy of graduation. Our team of board-certified physicians, nurses, medical students, and dentists had arrived a week earlier to Thomazeau, Haiti, a community about 20 miles north of Port-au-Prince along the banks of Lake Azuei. Situated in the town’s community center, we partnered with Haitian doctors and Haitian-American nurses to care for 1,000+ adults and children that week, most of whom had straightforward complaints such as generalized aches, gastritis, and rashes.

As we were closing, a young woman in a red T-shirt and jeans walked in, noticeably weak, and holding onto her male companion. She relayed that she had been vomiting and fainted. Her temperature measured 99.2°F, but she felt much warmer and her pulse was fast. With limited resources, we could not perform even basic laboratory or diagnostic testing. We relied on our histories and intuition for “sick” or “not sick.” She struggled to even hold up her head. She was undeniably ill, more so than we first appreciated.

Her left palm revealed irregularly spaced sutures entangled with dirt and leaves. She reported that they were placed 15 days earlier after cutting her hand on glass. The edges of her wound were indurated and had become more painful in recent days. We started ceftriaxone and hung normal saline on a barren pole, using manual pressure bagging to increase flow. It became clear to us and our attending physicians that she had sepsis and she needed an incision and drainage procedure.

With sunset approaching, the natural light flowing into the clinic was fading although it was still too early to turn on the generators. We moved her onto a wooden student’s desk and prepared the surgical field under a flashlight and a headlamp. After applying a topical anesthetic cream and sterilizing the palm, a long, shallow incision was made. With forceps, we searched for a foreign body but only found pieces of clotted blood mixed with dirt. Was this the nidus of her infection? Given no other source, we presumed so. The wound was irrigated, and then her hand was dressed and wrapped.

Though she was still tachypneic, tachycardic, and warm to touch, her blood pressure was deceptively normal. She was talking, moving, and urinating. Feeling reassured, we went to the second floor of the community center to retrieve dinner for her and her companion. As we were preparing plates of rice, beans, boiled potatoes, and plantains, our colleague alerted us that she had decompensated and we rushed down to find her diaphoretic and shivering. Multiple measurements confirmed her blood pressure to be 80s/30s and a thermometer registered her temperature at 102.5°F.

An impromptu conference with lead physicians and organization leaders was held at bedside. No local hospital was open. The nearest hospital was in Port-au-Prince, but it may take hours to complete registration and be seen. Was she stable enough to attempt it? We didn’t think so and concluded that she would remain with us.

We took turns keeping watch in the expansive yet empty ground floor of the community center, our makeshift clinic turned into a makeshift hospital lit by a flashlight. Her friend restlessly dozed on a nearby table, awakening whenever we attended to her care.

The peaceful, pitch-black night would occasionally be punctuated by the passing sound and light of a motorcycle. In the silence, our thoughts cycled between anxiety and curiosity. Should we have escalated care? What would we do if she stops breathing?

Alone with her, we wondered about her life. She did not speak English, we knew only a few phrases in Haitian Creole, and our interpreters were soundly asleep. What was she thinking? What were her worries, struggles, and anxieties? Did she grasp what was happening and how terribly sick she was?

Without the burden of returning pages or placing orders, we had this rare opportunity to spend uninterrupted hours with our patient. It allowed us to consider questions that we otherwise would not necessarily contemplate.

We knew that Haiti was the poorest country in the Western Hemisphere. Once a fertile colony, Haiti was saddled with inordinate debt from indemnity payments to France when it declared independence. Prosperity has been hindered by high-interest loans, chronic governmental corruption, political instability, and a negative trade balance. Haiti’s deforested lands stand in stark contrast to its neighbor, the Dominican Republic.

Two years after the catastrophic magnitude 7.0 earthquake in 2010 rattled its already weak infrastructure, we saw reminders of its enduring damage in the makeshift tent cities in Port-au-Prince and UN peacekeepers in the streets. Per the United Nations’ Human Development report, 59% live below the poverty line, 39% are illiterate, only 12% have internet access, and Haitians’ life expectancy of 63.7 years ranks 186th in the world.

As we quietly monitored, we wondered how this affected her. We had learned about social determinants of health and their impact on health outcomes. We wondered about her education and literacy. Did she have a stable home, enough money, or food security?

We could only imagine what her day was like. We saw most people walking to their destination. We saw children carrying 5-gallon water buckets on their head. We saw more cooking fires illuminating the night than homes with electric lights. Much of the commerce occurred with individuals peddling items rather than in grocery stores or pharmacies. We knew this was a limited snapshot. In that moment, we wished we could have heard her life story. We never got the chance as she woke up in the morning feeling better and, after eating a full breakfast, walked out of the clinic with a course of oral antibiotics.

Our worldviews have been so heavily influenced by our childhoods, our social circles, and our neighborhood. Our medical school prided itself on having a state-wide campus covering cities and rural towns across Wisconsin. We made house calls to Amish families, treated uninsured patients at a free clinic in a church basement, and cared for stereotypically stoic Midwestern farmers. The structured and efficient patient interview we learned in medical school missed capturing our patients’ lives and worries. We have recounted this experience numerous times over the years. As we proceeded through training, this experience was juxtaposed against the hurried interactions that filled our days. It continues to highlight the privilege of this unrushed moment.

Spending time with our patients has allowed us to see the world through a unique lens. Their perspective is informed by their lived experiences. Patients who have lived elsewhere, have had different occupations, religious views, political leanings, and have lived through a different generation. Although their beliefs and worries appeared foreign at first glance, we developed a richer understanding of our society.

